# Acknowledgment to the Reviewers of *Muscles* in 2022

**DOI:** 10.3390/muscles2010004

**Published:** 2023-01-16

**Authors:** Muscles Editorial Office

**Affiliations:** MDPI AG, St. Alban-Anlage 66, 4052 Basel, Switzerland

High-quality academic publishing is built on rigorous peer review. *Muscles* was able to uphold its high standards for published papers due to the outstanding efforts of our reviewers. Thanks to the efforts of our reviewers in 2022, the median time to first decision was 21 days and the median time to publication was 52 days. Regardless of whether the articles they examined were ultimately published, the editors would like to express their appreciation and thank the following reviewers for the time and dedication that they have shown *Muscles*:

Andrea BrancaccioLuisa GorzaAngelo MontanaLuz Berenice López-HernándezAnton SobinovMałgorzata ZimowskaCatherine SaenzMarcelo RugieroChengyi SunMaria CamposChristophe PraudMarta Seco-CerveraDaniel NewmireMatias MosqueiraDaniela RossiMoacir MarocoloDanny LumNatalia MerkulyevaDidier F. PisaniNatalia UgarovaEleni KarligiotouNathaniel J. SzewczykEshwar Reddy TammineniNejc UmekFrank E. NelsonNicolae GicaGerasimos TerzisNikunj A. BhagatGillian Sandra Butler-BrowneNuno PimentaGlen PyleOlga KarpichevaGregory CantrellPaulo Alexandre S. Armada da SilvaGuglielmo DurantiPiotr GawdaHarukaze YatsugiPiotr KaczkaIurii S. StafeevPrasanna KattiJan BilskiRobert Andrew ShanelyJernej RoskerSamo RauterJoão Batista Ferreira‐JuniorSatoshi KametakaJosé Peña-AmaroSimeon P. CairnsJoseph W. GordonSvein-Ole MikalsenJoshua P. BaileyThomas O. KragKairi HayashiTibor HortobagyiLászló DuxXu ZhangLaura SalvadoriYurii BorovikovLeland BarkerYusheng LiangLeonid V. Zingman


